# ﻿Three new species of *Nigrograna* (Dothideomycetes, Pleosporales) associated with Arabica coffee from Yunnan Province, China

**DOI:** 10.3897/mycokeys.94.95751

**Published:** 2022-12-12

**Authors:** Li Lu, Samantha C. Karunarathna, Dong-qin Dai, Ruvishika S. Jayawardena, Nakarin Suwannarach, Saowaluck Tibpromma

**Affiliations:** 1 Center for Yunnan Plateau Biological Resources Protection and Utilization, College of Biological Resource and Food Engineering, Qujing Normal University, Qujing, Yunnan 655011, China; 2 Center of Excellence in Fungal Research, Mae Fah Luang University, Chiang Rai 57100, Thailand; 3 School of Science, Mae Fah Luang University, Chiang Rai 57100, Thailand; 4 Research Center of Microbial Diversity and Sustainable Utilization, Faculty of Science, Chiang Mai University, Chiang Mai 50200, Thailand

**Keywords:** 3 new taxa, *Coffeaarabica*, Nigrogranaceae, phylogeny, saprobic fungi, taxonomy

## Abstract

Coffee is one of the most important cash crops in Yunnan Province, China. Yunnan is ranked as the biggest producer of high-quality coffee in China. During surveys of microfungi from coffee plantations in Yunnan, six fungal strains that resemble Nigrogranaceae were collected. Multi-gene analyses of a combined SSU-LSU-ITS-*rpb*2-*tef*1-α sequence data matrix were used to infer the phylogenetic position of the new species in *Nigrograna* while morphological characteristics were used to deduce the taxonomic position of the new species. Six fungal strains isolated from decaying branches of *Coffeaarabica* represent three new saprobic species in *Nigrograna*. The three new species, *N.asexualis*, *N.coffeae*, and *N.puerensis*, are described with full (macro and micro characteristics) descriptions, illustrations, and a phylogenetic tree that shows the phylogenetic position of new taxa.

## ﻿Introduction

Coffee (*Coffea* L.) was first planted in Yunnan Province, China in 1982 (Zhang et al. 2014). To date, about 170 varieties of coffee (Global Biodiversity Information Facility database (GBIF), available at: https://www.gbif.org/species/2895315 (accessed on 07 November 2022)) are available in the world, of which *Coffeaarabica* L. is the most popular coffee accounting for 75% of the world’s production, while 25% is provided by *C.canephora* Pierre ex A. Froehner, and less than 1% by *C.liberica* W. Bull and other varieties (Sharma 2020). The coffee production in Yunnan Province is approximately 90% of China’s total coffee production (Neilson and Wang 2019), while Pu’er is the largest coffee planting area in Yunnan, in terms of the highest yield and the best quality (Li 2014).

Fungal diversity is highly uncertain; the current estimated numbers are between 1.5 to 12 million, of which about 150,000 species have been named and classified (Hawksworth and Lücking 2017; Hyde et al. 2020; Bhunjun et al. 2022). Fungi are important organisms in terrestrial and aquatic ecosystems that are involved in the decomposition and nutrient cycling of dead plant material (Hyde et al. 2020; Bhunjun et al. 2022; Phukhamsakda et al. 2022). Also, saprobic fungi play vital roles in soil food chains, decomposition of plant, and animal materials, and solubilization of phosphorous (Dighton 2003; Pandey et al. 2008). However, coffee saprobic fungi have been poorly investigated (Arias and Abarca 2014; Lu et al. 2022a). Coffee saprobic fungi are distributed in 15 orders, and among them, Pleosporales Luttr. is the most common order (Lu et al. 2022a).

Pleosporales, belonging to Dothideomycetes O.E. Erikss. & Winka, was first proposed by Luttrell (1955), and later it was formally established by Barr (1987). In 2021, it consists of 91 families and 614 genera as the largest order (Hongsanan et al. 2020a; Wijayawardene et al. 2022). They are distributed in terrestrial and aquatic habitats (Zhang et al. 2008; Jiang et al. 2021). The members of Pleosporales are characterized by perithecioid and ostiolar ascomata, with or without periphyses, presence of cellular pseudoparaphyses, bitunicate, with ocular chambers or apical ring asci, various shapes of ascospores, with pigmentation and septation, and sheath present or absent (Zhang et al. 2012; Tennakoon et al. 2021; Yang et al. 2022).

Nigrogranaceae Jaklitsch & Voglmayr (Pleosporales) was proposed as a new family by Jaklitsch and Voglmayr (2016) to accommodate *Nigrograna* Gruyter, Verkley & Crous as the type genus. Liu et al. (2017) estimated that the divergence time of Nigrogranaceae is around 79 (44–124) Mya in crown age and 131 (86–180) Mya in stem age. Nigrogranaceae is monotypic, and they exist as endophytic, human pathogenic, and saprobic lifestyles (Hongsanan et al. 2020b; Zhang et al. 2020; Boonmee et al. 2021). The sexual morph of Nigrogranaceae is characterized by globose and black, ostiolar, clavate, and fissitunicate ascomata, with a short stipe and asci with a knob-like base, fusoid to narrowly ellipsoid, septate, and smooth or faintly verruculose ascospores (Jaklitsch and Voglmayr 2016). The asexual morph is characterized by pycnidia similar to ascomata, filiform and branched conidiophores, ampulliform or lageniform phialides, rod-like to ellipsoid, and hyaline or sub-hyaline conidia (Jaklitsch and Voglmayr 2016).

*Nigrograna* was introduced by de Gruyter (2012) with *N.mackinnonii* (Borelli) Gruyter, Verkley & Crous (basionym: *Pyrenochaetamackinnonii* Borelli) as the type species. *Pyrenochaetamackinnonii* was reported from a mycetoma patient by Borelli (1976), but it was found to be remote from the generic type species *P.nobilis* De Not. (de Gruyter et al. 2010, 2013). Since it was not possible to determine which family in Pleosporales*P.mackinnonii* belongs to, only the new genus *Nigrograna* was introduced to accommodate *P.mackinnonii* and named as *N.mackinnonii* (de Gruyter 2012). Later, *Nigrograna* was used as a synonym of *Biatriospora* K.D. Hyde & Borse, as *N.mackinnonii* is phylogenetically closely related to the type species of *Biatriospora* (*B.marina* K.D. Hyde & Borse) (Ahmed et al. 2014), while Hongsanan et al. (2020a) treated *Biatriospora* and *Nigrograna* as two separate genera. In 2022, *Nigrograna* represents 20 epithets listed in Index Fungorum (2022), and the members have been reported as saprobic, human pathogenic, and endophytic worldwide (Kolařík 2018; Zhao et al. 2018), showing a wide range of hosts (marine and terrestrial habitats) (Hyde et al. 2017; Tibpromma et al. 2017; Dayarathne et al. 2020). The sexual morph of *Nigrograna* is characterized by globose to subglobose and black ascomata, with ostiolar, two-layered peridium, clavate and fissitunicate asci, fusoid to narrowly ellipsoid, straight or curved, septate, and smooth or verruculose ascospores (Jaklitsch and Voglmayr 2016; Zhang et al. 2020). Asexual morph is characterized by globose to subglobose or pyriform pycnidia, filiform and branched conidiophores, hyaline, phialidic, discrete conidiogenous cells, sub-hyaline, aseptate and ellipsoidal conidia (de Gruyter 2012; Jaklitsch and Voglmayr 2016).

In this study, three saprobic *Nigrograna* were collected from *Coffeaarabica* branches in Yunnan Province, China. One species was isolated as an asexual morph (*N.asexualis*), while the other two isolated as sexual morphs (*N.coffeae*, *N.puerensis*) are illustrated and described as new species based on morphology and multi-gene phylogenetic analyses and are compared with closely related taxa.

## ﻿Materials and methods

### ﻿Collection, morphology and isolation

Coffee branch samples were collected from coffee plantations in Pu’er and Xishuangbanna, Yunnan Province, China. Specimens were put in plastic bags and taken to the mycology laboratory at Qujing Normal University. The vertical sections of fruiting structures were made for microscope studies and photomicrography. Micro-morphological characteristics were observed using a Leica DM2500 compound microscope and photographed with a Leica DMC4500 camera fitted onto the microscope. Color codes in the manuscript followed colorhexa (https://www.colorhexa.com). The measurements were processed in Tarosoft (R) Image Frame Work v. 0.9.7, and photographic plates were made in Adobe Photoshop CC 2018. Single spore isolation was carried out following Senanayake et al. (2020). Herbarium specimens were deposited at Zhongkai University of Agriculture and Engineering (**ZHKU**), while the living cultures growing on potato dextrose agar (**PDA**) were deposited at the culture collection of Zhongkai University of Agriculture and Engineering (**ZHKUCC**). Faces of fungi (**FoF**) numbers and Index Fungorum (**IF**) numbers were obtained as explained in Jayasiri et al. (2015) and Index Fungorum (2022).

### ﻿DNA extraction and PCR amplification

Genomic DNA was extracted from the fresh fungal mycelia which were grown on PDA for about two weeks, using Biospin Fungus Genomic DNA Extraction Kit–BSC14S1 (BioFlux, China) following the manufacturer’s instructions. Lu et al. (2021) was followed for the Polymerase Chain Reaction (PCR). Five partial gene regions were used in this study viz. the internal transcribed spacer (ITS) region was amplified with the primers ITS4 and ITS5 (White et al. 1990), the 18 s small subunit (SSU) region was amplified by primers NS1 and NS4 (White et al. 1990), the nuclear ribosomal 28 s large subunit (LSU) region was amplified by the primers LROR and LR5 (Vilgalys and Hester 1990), the partial RNA polymerase II subunit (rpb2) region was amplified with the primers RPB2-5F and RPB2-7cR (Liu et al. 1999), and the partial translation elongation factor 1-alpha (*tef1-α*) gene was amplified with the primers EF1-983F and 2218R (Rehner and Buckley 2005). Lu et al. (2022b) was followed for the amplification reactions of different primers. Amplified PCR products were sent to Sango Biotechnology Co., Ltd. (Shanghai, China) for sequencing. All sequences generated in this study were deposited in GenBank (Table 1).

**Table 1.** Taxa names, strain numbers, and corresponding GenBank accession numbers of the taxa used in the phylogenetic analyses. Newly generated sequences in this study are indicated in bold. The type species are noted with ^T^ after the species name, while NA indicates the unavailability of data.

Taxon	Strain numbers	ITS	LSU	*rpb*2	SSU	*tef*1-α
*Cyclothyriellarubronotata* (Berk. & Broome) Jaklitsch & Voglmayr ^T^	CBS 141486	KX650544	KX650519	NA	KX650507	KX650574
*Cyclothyriellarubronotata*	CBS 419.85	NA	GU349002	GU301875	NA	GU371728
*Nigrogranaantibiotica* (M. Kolařík & A. Kubátová) M. Kolařík ^T^	CCF 4378	JX570932	KF925327	NA	KF925328	JX570934
*Nigrogranaantibiotica*	CCF 4998	LT221894	NA	LT221895	NA	NA
*Nigrogranaaquatica* W. Dong, H. Zhang & K.D. Hyde ^T^	MFLUCC 14-1178	MF399065	MF415392	NA	MF415394	MF498582
*Nigrogranaaquatica*	MFLUCC 17-2318	MT627705	MN913705	NA	NA	NA
***Nigrogranaasexualis*** T	**ZHKUCC 22**-**0214**	**OP450965**	**OP450971**	**OP432241**	**OP450979**	**OP432245**
***Nigrogranaasexualis***	**ZHKUCC 22**-**0215**	**OP450966**	**OP450972**	**OP432242**	**OP450980**	**OP432246**
*Nigrogranacangshanensis* Z.L. Luo, H.Y. Su & K.D. Hyde ^T^	MFLUCC 15-0253	KY511063	KY511064	NA	KY511065	NA
*Nigrogranacarollii* M. Kolařík ^T^	CCF 4484	LN626657	LN626682	LN626662	LN626674	LN626668
*Nigrogranachromolaenae* Mapook & K.D. Hyde ^T^	MFLUCC 17-1437	MT214379	MT214473	NA	NA	MT235801
***Nigrogranacoffeae*** ^T^	**ZHKUCC 22**-**0210**	**OP450967**	**OP450973**	**OP432243**	**OP450981**	**OP432247**
***Nigrogranacoffeae***	**ZHKUCC 22**-**0211**	**OP450968**	**OP450974**	**OP432244**	**OP450982**	**OP432248**
*Nigrogranafuscidula* (Sacc.) Jaklitsch & Voglmayr ^T^	CBS 141556	KX650550	NA	NA	NA	KX650525
*Nigrogranafuscidula*	CBS 141476	KX650547	NA	KX650576	KX650509	KX650522
*Nigrogranafuscidula*	MF1a	KX650548	NA	NA	NA	KX650523
*Nigrogranafuscidula*	MF3	KX650549	NA	NA	NA	KX650524
*Nigrogranahydei* J.F. Zhang, J.K. Liu & Z.Y. Liu ^T^	GZCC 19-0050	MN387225	MN387227	NA	NA	MN389249
*Nigrogranaimpatientis* J.F. Zhang, J.K. Liu & Z.Y. Liu ^T^	GZCC 19-0042	MN387226	MN387228	NA	NA	MN389250
*Nigrogranajinghongensis* Wanas. & K.D. Hyde ^T^	KUMUCC 21-0035	MZ493303	MZ493317	MZ508421	MZ493289	MZ508412
*Nigrogranajinghongensis*	KUMUCC 21-0036	MZ493304	MZ493318	MZ508422	MZ493290	MZ508413
*Nigrogranakunmingensis* T.Y. Du & Tibpromma ^T^	ZHKUCC 22-0242	OP456214	OP456379	NA	OP456382	OP471608
*Nigrogranakunmingensis*	ZHKUCC 22-0243	OP484334	OP456380	NA	OP456383	OP471609
*Nigrogranalocuta-pollinis* F. Liu & L. Cai ^T^	CGMCC 3.18784	MF939601	MF939583	MF939610	NA	MF939613
*Nigrogranalocuta-pollinis*	LC11690	MF939603	MF939584	MF939611	NA	MF939614
*Nigrogranamackinnonii* ^T^	CBS 674.75	KF015654	KF015612	KF015703	GQ387552	KF407986
*Nigrogranamackinnonii*	E5202H	JX264157	KJ605422	JX264156	JX264155	JX264154
*Nigrogranamackinnonii*	E9303e	JN545759	LN626681	LN626666	LN626678	LN626673
*Nigrogranamagnoliae* Wanas. ^T^	MFLUCC 20-0020	MT159628	MT159622	MT159611	MT159634	MT159605
*Nigrogranamagnoliae*	GZCC 17-0057	MF399066	MF415393	NA	MF415395	MF498583
*Nigrogranamagnoliae*	MFLUCC 20-0021	MT159629	MT159623	MT159612	MT159635	MT159606
*Nigrogranamycophila* Jaklitsch, Friebes & Voglmayr ^T^	CBS 141478	KX650553	NA	NA	NA	KX650526
*Nigrogranamycophila*	CBS 141483	KX650555	NA	KX650577	KX650510	KX650528
*Nigrogranamycophila*	MF6	KX650554	NA	NA	NA	KX650527
*Nigrogrananorvegica* Jaklitsch & Voglmayr ^T^	CBS 141485	KX650556	NA	KX650578	KX650511	NA
*Nigrogranaobliqua* Jaklitsch & Voglmayr ^T^	CBS 141477	KX650560	NA	KX650580	NA	KX650531
*Nigrogranaobliqua*	CBS 141475	KX650558	NA	KX650579	KX650512	KX650530
*Nigrogranaobliqua*	MRP	KX650561	NA	KX650581	NA	KX650532
*Nigrogranaperuviensis* (M. Kolařík & R. Gazis) M. Kolařík ^T^	CCF 4485	LN626658	LN626683	LN626665	LN626677	LN626671
***Nigrogranapuerensis*** ^T^	**ZHKUCC 22-0212**	**OP450969**	**OP450975**	**NA**	**OP450983**	**OP432249**
***Nigrogranapuerensis***	**ZHKUCC 22-0213**	**OP450970**	**OP450976**	**NA**	**OP450984**	**OP432250**
*Nigrogranarhizophorae* Dayar., E.B.G. Jones & K.D. Hyde ^T^	MFLUCC 18-0397	MN047085	NA	MN431489	NA	MN077064
*Nigrogranarhizophorae*	MFLU 19-1234	NA	MN017845	MN431490	NA	MN077063
*Nigrogranasamueliana* Devadatha, V.V. Sarma & E.B.G. Jones ^T^	NFCCI-4383	MK358817	MK358812	MK330939	MK358810	MK330937
*Nigrogranathymi* Mapook, Camporesi & K.D. Hyde ^T^	MFLUCC 14-1096	KY775576	KY775573	NA	KY775574	KY775578
*Nigrogranayasuniana* M. Kolařík ^T^	YU.101026	HQ108005	LN626684	LN626664	LN626676	LN626670
*Occultibambusabambusae* D.Q. Dai & K.D. Hyde ^T^	MFLUCC 13-0855	KU940123	KU863112	KU940170	NA	KU940193
*Occultibambusafusispora* Phookamsak, D.Q. Dai & K.D. Hyde	MFLUCC 11-0127	MZ329036	MZ325466	MZ329032	MZ329028	MZ325469
*Occultibambusapustula* D.Q. Dai & K.D. Hyde ^T^	MFLUCC 11-0502	KU940126	KU863115	NA	NA	NA
*Paradictyoarthriniumdiffractum* Matsush.	MFLUCC13-0466	KP744455	NA	KP744498	NA	NA
*Paradictyoarthriniumtectonicola* Doilom & K.D. Hyde ^T^	MFLUCC 13-0465	KP744456	NA	KP744500	KP753961	KX437763
*Seriascomadidymosporum* Phookamsak, D.Q. Dai, Karun. & K.D. Hyde ^T^	MFLUCC 11-0179	KU940127	KU940196	KU863116	NA	KU940173
*Seriascomahonghense* H.B. Jiang, Phookamsak & K.D. Hyde ^T^	KUMCC 21-0021	MZ329039	MZ325468	MZ329035	NA	MZ325470
*Versicolorisporiumtriseptatum* Sat. Hatak., Kaz. Tanaka & Y. Harada ^T^	HHUF 28815	NR_119392	NA	NG_042318	NG_060995	NA

### ﻿Phylogenetic analyses

Phylogenetic analyses of the aligned sequences referred to Dissanayake et al. (2020). Newly generated reverse and forward sequences were assembled with Geneious program (9.1.2) and the preliminary identification was done by the BLASTn search in NCBI (https://www.ncbi.nlm.nih.gov). Additional highly similar sequences were downloaded from GenBank (https://www.ncbi.nlm.nih.gov/genbank/) based on the BLASTn results and recent publications. Single-gene sequence alignments were made in MAFFT v. 7 (http://mafft.cbrc.jp/alignment/server/), edited in trimAl v1.2 (http://trimal.cgenomics.org), and multi-gene alignments were made by Sequence Matrix program (1.7.8) (Vaidya et al. 2011). The sequence datasets used to build the phylogenetic trees are shown in Table 1.

Phylogenetic analyses were conducted with maximum likelihood (ML) and Bayesian inference (BI) algorithms on the CIPRES Science Gateway portal (https://www.phylo.org/) (Miller et al. 2012). The ML tree was run with RAxML-HPC v.8 on XSEDE (Stamatakis 2014), and GTRGAMMA substitution model with 1000 bootstrap iterations. The BI tree was run with MrBayes on XSEDE (3.2.7a) (Ronquist et al. 2012). MrModeltest 2.2 (Nylander 2004) and PAUP v. 4.0b10 (Ronquist and Huelsenbeck 2003) were used to evaluate the best models of evolution, the evolutionary model of SYM+I+G substitution model was selected for LSU, HKY+I+G substitution model was selected for SSU, and GTR+I+G substitution model was selected for ITS, *rpb*2 and *tef*1-α. Six simultaneous Markov Chains were run for two million generations and trees were sampled at every 200^th^ generation (resulting in 10,000 trees), and these chains stopped when all convergences met and the standard deviation fell below 0.01. All resulting trees were plotted using FigTree v. 1.4.0 (Rambaut 2014) and the layout of the trees was made by Microsoft Office PowerPoint 2020.

## ﻿Results

### ﻿Phylogenetic analyses

Three new species formed a distinct clade in *Nigrograna* with strong statistical support (*N.coffeae* and *N.puerensis*ML = 100%, BIPP = 1.00, and *N.asexualis*ML = 68%, BIPP = 0.97). Multi-locus data (SSU, LSU, ITS, *rpb*2 and *tef*1-α) composed of 54 strains (Table 1), and *Cyclothyriellarubronotata* strains CBS 141486 and CBS 419.85 were used as the outgroup taxa. A total of 4485 characters were fed to the phylogenetic analysis after alignment, 1–1047 (SSU), 1048–1956 (LSU), 1957–2477 (ITS), 2478–3510 (*rpb*2) and 3511–4485 (*tef*1-α). The topology of the phylogenetic tree generated by the ML method was highly similar to that by BI, and therefore it was chosen to represent the evolutionary history of *Nigrograna*.

**Figure 1.**
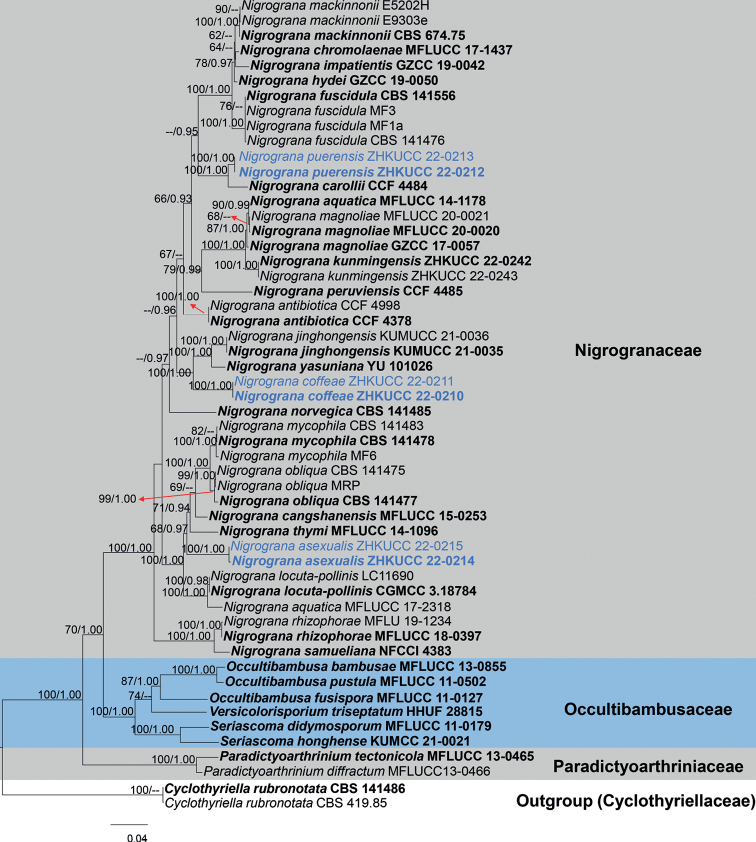
The maximum-likelihood phylogram of *Nigrograna* based on a combined SSU, LSU, ITS, *rpb*2 and *tef*1-α sequence dataset with *Cyclothyriellarubronotata* CBS 141486 and CBS 419.85 as the outgroup taxa (Dayarathne et al. 2020). The maximum-likelihood bootstrap values (ML ≥ 60%, left) and Bayesian Inference Posterior Probability values (BIPP ≥ 0.90, right) are shown above the nodes. Strains derived from the current study are in blue, while type strains are in bold.

The ML analysis of the combined dataset yielded a best-scoring tree with a final ML optimization likelihood value of -23091.568105. The alignment has 1495 distinct alignment patterns, with 33.58% completely undetermined characters and gaps. Parameters for the GTR + I + G model of the combined SSU, LSU, ITS, *rpb*2 and *tef*1-α were as follows: estimated base frequencies A = 0.247145, C = 0.250645, G = 0.263985, T = 0.238225; substitution rates AC = 1.810004, AG = 4.475190, AT = 1.758134, CG = 1.340389, CT = 10.583215, GT = 1.000; gamma distribution shape parameter α = 0.167006. The phylogenetic tree resulting from RAxML analysis is shown in Fig. 1.

### ﻿Taxonomy

#### ﻿ Nigrograna coffeae

Taxon classificationFungiPleosporalesBiatriosporaceae

L. Lu & Tibpromma sp. nov.

EB69069B-B814-520B-BFD4-4E61265EAB85

Index Fungorum number: IF559425

Facesoffungi Number: FoF12765

Fig. 2

##### Etymology.

Species epithet refers to the host genus “*Coffea*” where the fungus was isolated.

##### Holotype.

ZHKU 22-0121.

##### Description.

***Saprobic*** on decaying branch of *Coffeaarabica*. **Sexual morph: *Ascomata*** 90–140 µm high, 140–200 μm wide (x̄ = 115 × 168 μm, n = 10), immersed, solitary, black spots on substrate, subglobose to oval, sometimes obpyriform, some with ostiolate. ***Peridium*** 10–15 µm wide, composed of 3–5 layers, hyaline to brown (#937463) cells of ***textura angularis***. ***Hamathecium*** 1.5–3 μm wide, composed of numerous, hyaline, filamentous, septate, branched, pseudoparaphyses. ***Asci*** 50–70 × 7–11 μm (x̄ = 58 × 9 μm, n = 20), 8-spored, bitunicate, fissitunicate, clavate to cylindric-clavate, short stalked, some with club-shape pedicel, apically rounded, with a small ocular chamber. ***Ascospores*** 12–16 × 4–5 μm, (x̄ = 14.4 × 4.6 μm, n = 30), overlapping uni- to bi-seriately arranged, fusiform, straight or slightly curved, hyaline when immature and become pale brown (#e1af33) to dark-brown (#6e5031) when mature, mostly 1-septate, few 2 or 3-septate, constricted at each septum, with obviously guttulate. **Asexual morph**: Undetermined.

##### Culture characteristics.

Ascospores germinated on PDA within 24 h and germ tubes arising from both ends. Colonies on PDA, reaching 4.5 cm diam. after two months of incubation at room temperature (22–26 °C), initially white (#f2f3f4) becoming grey (#bbbeb2) to dark brown (#6e5031) at maturity, dense, circular, slightly raised, smooth surface, radially fimbriate at the edge, reverse dark green (#3a4543) to brown (#937463).

##### Material examined.

Pu’wen Town, Xishuangbanna, Yunnan Province, China, on a decaying branch of *Coffeaarabica*, (22°31'18"N, 101°2'44"E, 856.89 m), 15 September 2021, LiLu, JHPW16 (ZHKU 22-0121, holotype), ZHKUCC 22-0210 = ZHKUCC 22-0211. GenBank number; ITS: OP450967, LSU: OP450973, *rpb*2: OP432243, SSU: OP450981, *tef*1-α: OP432247 (ZHKUCC 22-0210, ex-type); ITS: OP450968, LSU: OP450974, *rpb*2: OP432244, SSU: OP450982, *tef*1-α: OP432248 (ZHKUCC 22-0211).

##### Notes.

Our phylogenetic analyses showed that *Nigrogranacoffeae* forms an independent clade (100% ML, 1.00 BIPP, Fig. 1), and is phylogenetically related to *N.yasuniana* and *N.jinghongensis*. *Nigrogranayasuniana* was reported as endophytes from *Conceveibaguianensis* Aubl. in Ecuador, but there were not enough morphological data, the comparison of base pairs in ITS showed 3.4% differences (15/433 bp), LSU showed 1.5% differences (12/812bp), SSU only showed 0.3% differences (3/1028 bp), *rpb*2 showed 14% differences (117/829 bp), and *tef*1-α showed 3.2% differences (31/954 bp) (Kolařík et al. 2017). *Nigrogranajinghongensis* was introduced as a saprobic fungus from woody litter in China, and our new isolate shares a similar size (12–16 × 4–5 μm *vs* 12–15 × 4–5.5 µm) and color (hyaline to dark brown *vs* yellowish-brown to brown) of ascospores with *N.jinghongensis* (Boonmee et al. 2021), but there are some significant differences in the size of the ascomata (90–140 µm high, 140–200 μm wide *vs* 300–400 µm high 220–300 μm wide) and the shape of ascospores (fusiform, straight or slightly curved *vs* ellipsoid) (Boonmee et al. 2021). Based on the sequence blast results, ITS, LSU and *rpb*2 gene sequences were similar to *Nigrograna* sp., with 97.5% (MZ270683), 98.4% (MK762716), and 86% (MZ508421) respectively, SSU was similar to *N.mycophila* with 99% (KX650510), and *tef*1-α was similar to *N.yasuniana* with 96.6% (LN626670). Therefore, we introduce our new isolate as a new species *N.coffeae* based on both morphological characteristics and phylogenetic analyses.

**Figure 2.**
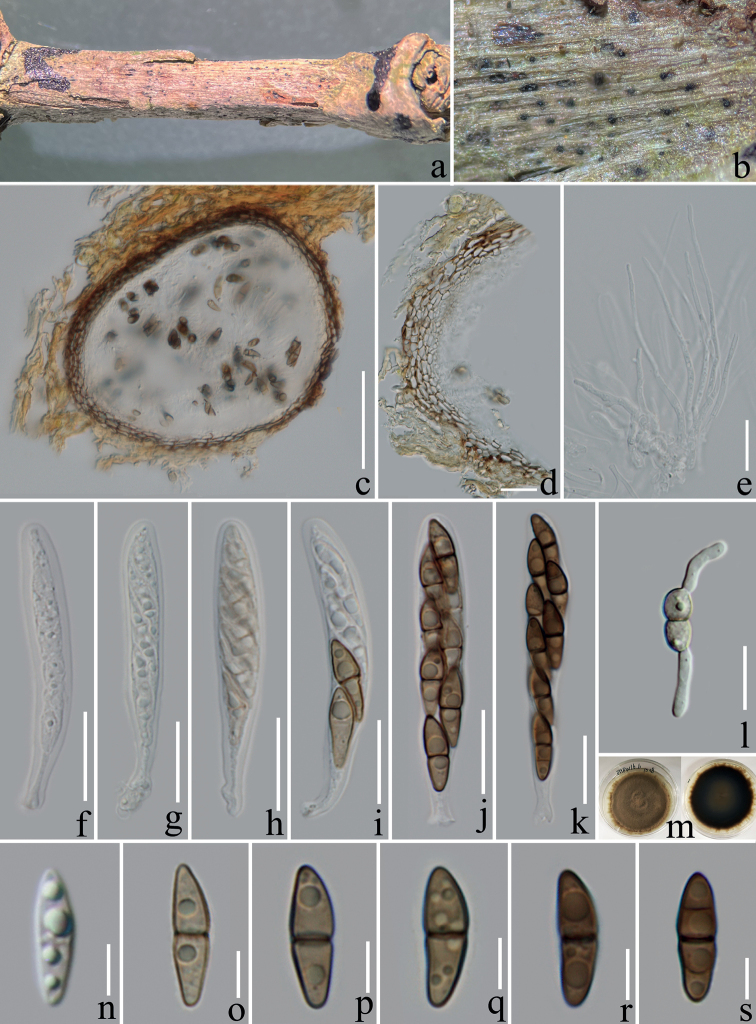
*Nigrogranacoffeae* (ZHKU 22-0121, holotype) **a, b** ascomata on the host substrate **c** a vertical section through an ascoma **d** peridium **e** hamathecium **f–k** asci **l** germinated ascospore **m** culture on pda from above and reverse **n–s** ascospores (arrows indicate the septa). Scale bars: 50 μm (**c**); 10 μm (**d–l**); 5 μm (**n–s**).

#### ﻿ Nigrograna puerensis

Taxon classificationFungiPleosporalesBiatriosporaceae

L. Lu & Tibpromma sp. nov.

1B95946C-F016-5EA6-988D-8E1715C78A93

Index Fungorum number: IF559426

Facesoffungi Number: FoF12766

Fig. 3

##### Etymology.

The specific epithet “*puerensis*” refers to the location Pu’er City, where the type species was collected.

##### Holotype.

ZHKU 22-0122.

##### Description.

***Saprobic*** on decaying branch of *Coffeaarabica*. **Sexual morph: *Ascomata*** 90–180 µm high, 90–150 μm wide (x̄ = 138 × 115 μm, n = 10), immersed, with only ostiolar necks visible on the host surface or erumpent, solitary, subglobose to ellipsoid, dark brown (#6e5031). ***Peridium*** 10–15 μm wide (x̄ = 13 μm, n = 15), outer layer consists of 2–3 layers of ***textura prismatica***, brown (#937463) and thick-walled cells, inner layer hyaline with thin-walled cells. ***Hamathecium*** composed of numerous, 1.5–2 µm wide (x̄ = 1.8 μm, n = 20), filamentous, hyaline, septate, pseudoparaphyse. ***Asci*** 50–80 × 8–11 μm (x̄ = 66 × 9.5 μm, n = 20), 8-spored, bitunicate, fissitunicate, cylindrical to clavate, short pedicellate, apically rounded, with poorly developed ocular chamber. ***Ascospores*** 15–18 × 4–5 μm, (x̄ = 16 × 4.5 μm, n = 30), uni- to bi-seriately arranged, fusoid, apical cell and basal cell acute, and apical cell slightly wider than basal cell, straight or slightly curved, 1-septate, constricted at septum, guttulate, hyaline to yellow-brownish (#daceb8) when young, brownish (#937463) when mature. **Asexual morph**: Undetermined.

##### Culture characteristics.

On PDA, colonies reached up to 4 cm diam. after two months at room temperature (22–26 °C). Colony dense, circular, slightly raised at the center, surface with white aerial mycelium, fluffy, with a serrate edge, grayish (#c9bfb3) to dark brown (#6e5031) from center to edge, reverse dark green (#3a4543) to dark brown (#6e5031).

##### Material examined.

Pu’er City, Yunnan Province, China, on a decaying branch of *Coffeaarabica*, (22°36'2"N, 101°0'59"E, 1016.43 m), 16 September 2021, LiLu, Puer 1-4 (ZHKU 22-0122, holotype), ZHKUCC 22-0212 = ZHKUCC 22-0213. GenBank number; ITS: OP450969, LSU: OP450975, SSU: OP450983, *tef*1-α: OP432249 (ZHKUCC 22-0212, ex-type); ITS: OP450970, LSU: OP450976, SSU: OP450984, *tef*1-α: OP432250 (ZHKUCC 22-0213).

##### Notes.

*Nigrogranapuerensis* clusters with *N.carollii* with significant statistical support from ML 100% and BIPP 1.00. In morphology, our new strains best fit *Nigrograna* by having immersed ascomata, clavate and short pedicellate asci, and pale to brown, fusoid to narrowly ellipsoid, and septate ascospores (Jaklitsch and Voglmayr 2016; Zhang et al. 2020). Blast search results of ITS, LSU and *tef*1-α sequence data revealed that our taxon (ZHKUCC 22-0212) is similar to *N.mackinnonii* (96% MZ270697, 99% KJ605422, and 95% LT797087 respectively), while the similarity of SSU sequence to *N.carollii* is as high as 99%. Based on nucleotide comparisons, our isolate (ZHKUCC 22-0212) differs from *N.carollii* (CCF 4484) by 9/490 bp (1.8%) in ITS, 2/222 bp (1%) in LSU, 2/1306 bp (0.2%) in SSU, and 10/530 bp (2%) in *tef*1-α. Unfortunately, for *N.carollii*, sufficient morphological data was not available to compare with our novel taxon which was isolated as an endophyte on living sapwood of wild *Heveabrasiliensis* Müll. Arg., and *N.mackinnonii* which was isolated as a human pathogen (de Gruyter 2012; Kolařík et al. 2017). In addition, the colony morphology of *N.carollii* on PDA is described as colonies plane, effuse, and light gray (Kolařík et al. 2017), while *N.puerensis* colony surface is seen as white aerial mycelium, fluffy, with a serrate edge, and grayish to dark brown from center to edge. Therefore, based on morphological and phylogenetic analyses, we introduce *N.puerensis* as a distinct new species.

**Figure 3.**
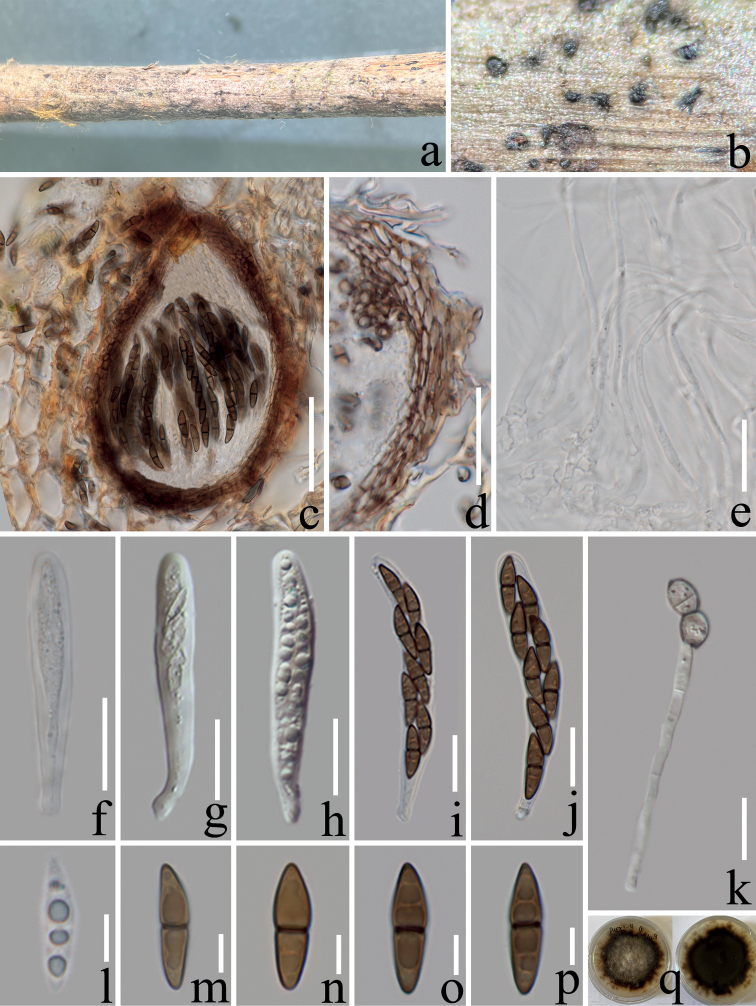
*Nigrogranapuerensis* (ZHKU 22-0122, holotype) **a, b** ascomata observed on host substrate **c** a vertical section through an ascoma **d** peridium **e** hamathecium **f–j** asci **k** germinated ascospore **l–p** ascospores **q** culture on PDA from above and reverse. Scale bars: 50 μm (**c**); 30 μm (**d**); 15 μm (**e–k**); 5 μm (**l–p**).

#### ﻿ Nigrograna asexualis

Taxon classificationFungiPleosporalesBiatriosporaceae

L. Lu & Tibpromma sp. nov.

6C59D4C7-E1AF-5732-BDD6-02F00DCC1623

Index Fungorum number: IF559427

Facesoffungi Number: FoF12767

Fig. 4

##### Etymology.

The species epithet ‘*asexualis*’ refers to the asexual morph.

##### Holotype.

ZHKU 22-0123.

##### Description.

***Saprobic*** on decaying branch of *Coffeaarabica.***Sexual morph**: Undetermined. **Asexual morph**: Coelomycetous. ***Pycnidia*** 100–230 µm high, 120–180 µm wide (x̄ = 156 × 144 µm, n = 10), globose to subglobose, or pyriform, immersed, solitary, unilocular, dark brown, papillate ostiole, appearing as black spots on host surface. ***Pycnidial wall*** 11–16 µm wide (x̄ = 14 µm, n = 15), brown (#937463), the wall with pseudoparenchymatous cells. ***Conidiophores*** arising from the pycnidial wall, up to 46 µm long and 3–4.4 µm wide (x̄ = 3.4 µm, n = 25), filiform, septate, hyaline, simple to sparsely branched, with pegs along one or two sides and solitary phialides terminally. ***Phialides*** 3–6 × 1–2 µm (x̄ = 4.5 × 1.5 µm, n = 15), variable in shape, phialidic, discrete, ampulliform-lageniform-subcylindrical. ***Conidia*** 5–6.5 × 3–4 µm (x̄ = 5.5 × 3.7 µm, n = 30), ellipsoidal, unicellular, aseptate with 1–2 granules, subhyaline, smooth-walled.

##### Culture characteristics.

Conidium germinated on PDA within 24 h. Colonies growing on PDA reaching 5 cm diam. after two months at room temperature (22–26 °C). Colony dense, circular, surface sparsely hairy, radially striate, with a fimbriate edge, yellowish (#eabf83) to pale brown (#e1af33) at the center and dark brown (#6e5031) at the margin, reverse dark brown (#6e5031).

##### Material examined.

Pu’er City, Yunnan Province, China, on a decaying branch of *Coffeaarabica*, (22°36'2"N, 101°0'59"E, 1016.43 m), 16 September 2021, LiLu, Puer 1-14 (ZHKU 22-0123, holotype), ZHKUCC 22-0214 = ZHKUCC 22-0215. GenBank number; ITS: OP450965, LSU: OP450971, *rpb*2: OP432241, SSU: OP450979, *tef*1-α: OP432245 (ZHKUCC 22-0214, ex-type); ITS: OP450966, LSU: OP450972, *rpb*2: OP432242, SSU: OP450980, *tef*1-α: OP432246 (ZHKUCC 22-0215).

##### Notes.

In multi-gene phylogeny, *Nigrogranaasexualis* formed a separate (68% ML, 0.97 BIPP) and distinct clade within *Nigrograna* (Fig. 1). Morphologically, *N.asexualis* conforms to the morphological characteristics of *Nigrograna* by having hyaline or subhyaline, long and branched conidiophores, solitary phialides, and aseptate, ellipsoidal or cylindrical conidia (Jaklitsch and Voglmayr 2016; Dayarathne et al. 2020; Wanasinghe et al. 2020). Blast results of the sequences show that ITS is similar to *N.fuscidula* with 89% (MH856004), and SSU is similar to *N.mycophila* with 99.8% (KX650510). *Nigrogranaasexualis* is different from *N.fuscidula* and *N.mycophila* by its ellipsoidal conidia, but the similarities of these three species are hyaline, 1-celled, smooth-walled conidia forming on philipides (Jaklitsch and Voglmayr 2016). The LSU and *rpb*2 sequences of our strain blast results are similar to *N.obliqua*, and the similarities are 98.9% (KX650560) and 87% (KX650579) respectively, but *N.obliqua* lacks the asexual morph (Jaklitsch and Voglmayr 2016). The *tef*1-α sequence of our strain is 95.8% (MF939615) similar to *N.locuta-pollinis*, which was isolated from hive-stored pollen of *Brassicacampestris* L. that lacks morphology (Zhao et al. 2018). Therefore, we introduce *N.asexualis* as a distinct new species from coffee in China.

**Figure 4.**
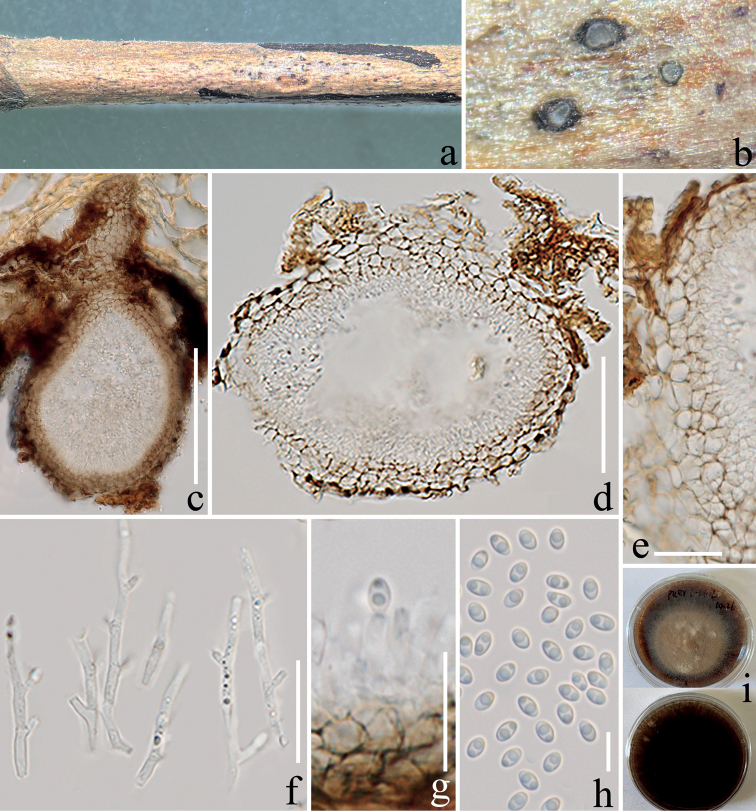
*Nigrogranaasexualis* (ZHKU 22-0123, holotype) **a, b** conidiomata on the host substrate **c, d** vertical sections of a conidioma **e** peridium **f, g** conidiophores with phialides **h** conidia **i** culture on PDA from above and reverse. Scale bars: 100 μm (**c**); 50 μm (**d**); 15 μm (**e**); 30 μm (**f**); 20 μm (**g**); 10 μm (**h**).

## ﻿Discussion

Members of *Nigrograna* are distributed worldwide in soil, wood, and other plant debris (Mapook et al. 2020), and the hotspots of *Nigrograna* are reported as Central and South America, where the taxa are also found as human pathogens (Kolařík 2018; Puing et al. 2020). To date, five *Nigrograna* species viz. *N.cangshanensis* (decaying wood, Yunnan), *N.jinghongensis* (dead woody litter, Yunnan), *N.kunmingensis* (dead twigs of *Gleditsiasinensis* Lam., Yunnan), *N.magnoliae* (living branches of *Magnoliadenudate* Desr., Yunnan), and *N.locuta-pollinis* (hive-stored pollen, Hubei) have been isolated from different hosts in China (Tibpromma et al. 2017; Zhao et al. 2018; Wanasinghe et al. 2020; Boonmee et al. 2021; Zhou et al. 2022). In this study, three new saprobic fungi were isolated from decaying branches of *Coffeaarabica* in Yunnan Province, China, and this is the first report of *Nigrograna* species from coffee.

Species of *Nigrograna* are morphologically very similar and overlapping, hence can be interpreted as cryptic species. Therefore, it is difficult to delimit the species based only on their morphological characteristics (Jaklitsch and Voglmayr 2016; Zhang et al. 2020). In our research, we found that *N.coffeae* and *N.puerensis* have similar morphology, but in phylogeny, they are distributed differently within *Nigrograna*. This confirms the view of Jaklitsch and Voglmayr (2016) that the gene sequences are important and crucial for the identification of taxa at the genus and the species level.

## Supplementary Material

XML Treatment for Nigrograna coffeae

XML Treatment for Nigrograna puerensis

XML Treatment for Nigrograna asexualis
